# Effects of 910 MHz Solid-State Microwave Cooking on the Quality Properties of Broccoli (*Brassica olearacea* L. var. *Italica* Plenck), Carrots (*Daucus carota* subsp. *Sativus*), and Red Peppers (*Capsicum annuum* L. cv. Kapya)

**DOI:** 10.3390/foods13213459

**Published:** 2024-10-29

**Authors:** Gönül Çavuşoğlu Kaplan, Ebru Fıratlıgil

**Affiliations:** Department of Food Engineering, Istanbul Technical University, Istanbul 34469, Turkey; ebruf@itu.edu.tr

**Keywords:** solid-state microwave cooking, microwave cooking, broccoli, red pepper, carrot

## Abstract

Domestic microwave ovens offer rapid cooking but face challenges such as non-uniform temperature distribution and hot spots. A novel solid-state heating system, which precisely controls microwave frequency and power, provides a promising alternative to traditional microwave ovens utilizing magnetron systems. This study compared the effects of solid-state microwave cooking on the quality of broccoli, red peppers, and carrots with those of traditional microwave and conventional cooking. The traditional microwave cooking used in this study operated at 2450 MHz, while the solid-state system functioned between 902 and 928 MHz. Weight loss was highest for conventional cooking, reaching a maximum of 34%, whereas microwave cooking resulted in a maximum of 11.65% and solid-state microwave cooking in 17.04%. The total phenolic content obtained through conventional cooking ranged between 61.58 and 116.51 mg GAE (gallic acid equivalents)/100 g dry basis, while microwave cooking resulted in a range of 88.04–110.92 mg, and solid-state microwave cooking achieved values between 76.14 and 122.91 mg. Furthermore, reductions in chlorophyll content were observed to be 68.2%, 25.6%, and 35.7% for conventional, microwave, and solid-state microwave cooking, respectively. Lycopene content after conventional cooking decreased to 224.73 mg/100 g dry basis, compared to 289.55 mg after microwave cooking and 242.94 mg after solid-state microwave cooking. β-carotene content showed a decrease of 14.5% in conventional cooking, while both microwave methods showed an increase of 14.7%. These results suggest that solid-state microwave cooking may have promising positive effects on food quality.

## 1. Introduction

Unlike conventional systems, dielectric cooking involves heating food through the interaction of alternating electromagnetic fields, dipoles, and ionic charges. High-frequency microwaves in the 300–300,000 MHz range are essential for dielectric heating methods in the food industry and in domestic appliances [1]. Commonly used frequency ranges in microwave heating applications are 902–928 MHz (centered at 915 MHz) and 2400–2500 MHz (centered at 2450 MHz), allocated to the industrial, scientific, and medical (ISM) radio bands. While 915 MHz is predominantly used in industrial microwave systems in the USA, 2450 MHz is employed in both domestic and industrial microwave ovens [2]. Domestic microwave ovens operating at a higher frequency based on magnetron systems offer a significant advantage in terms of fast cooking. However, the output frequency of the magnetron is not exactly 2450 MHz; it spans a broad range and is affected by factors such as the type and position of the food. For this reason, they also have disadvantages, such as a non-uniform temperature distribution resulting in the formation of hot spots in cooked food, which affect food quality and safety [3]. Recently, a novel cooking technique was developed to address these issues in traditional domestic microwave ovens. This solid-state heating system involves high-power amplifiers instead of magnetrons and provides various advantages, including frequency control and feedback mechanisms enabling dynamic cooking conditions [4]. The precise control of the microwave frequency, phase, and power provides an opportunity to influence the microwave field present within the cavity. Additionally, recent findings indicate that this new approach, which enables cooking at a precise frequency within a narrow band, offers greater stability and accuracy compared to magnetrons. Additionally, the controlled range of frequency variations improves both heating uniformity and energy efficiency [3,4,5]. Solid-state heating systems can assess feedback from forward and reflected power levels, which enables monitoring and modifying the energy load applied to food based on the changing dielectric and structural properties of the food during cooking. This system provides dynamic and efficient cooking by matching the frequency point of each food item with a unique reflected power [6]. Owing to these advantages, this technology holds great potential for improving various aspects of food processing in the industry and addressing key challenges [4].

Despite these advantages, research on the household use of solid-state heating technology remains limited. In recent years, studies have been conducted on the effects of this technology on homogeneity and energy efficiency. However, the impact of microwave frequency ranges permitted within regulatory limits on food quality is still unclear. In this study, vegetables were selected because they represent a food group where healthy cooking techniques are particularly important. Additionally, broccoli, red pepper, and carrots were chosen due to their distinct color and texture profiles, allowing for a broader evaluation of the cooking effects across different vegetable types. We determined the effects of solid-state microwave on their quality properties. Cooking experiments were conducted using a solid-state microwave cooking setup operating in the 902–928 MHz range. The results were compared with those obtained with traditional microwave ovens operating at a higher frequency (2450 MHz) and conventional cooking in a domestic oven. Various parameters were evaluated to assess the cooking quality, including the weight loss, moisture content, pH, soluble solids, color, texture, total phenolics, vitamin C, chlorophyll, lycopene, and beta-carotene content.

## 2. Materials and Methods

### 2.1. Materials

Fresh broccoli (*Brassica olearacea* L. var. *Italica* Plenck), carrots (*Daucus carota* subsp. *Sativus*), and red pepper (*Capsicum annuum* L. cv. Kapya) were purchased from a local market in Istanbul, Turkey. Each vegetable was prepared by removing the inedible parts and cutting them into homogeneous pieces.

### 2.2. Solid-State Microwave System

**Figure 1:**
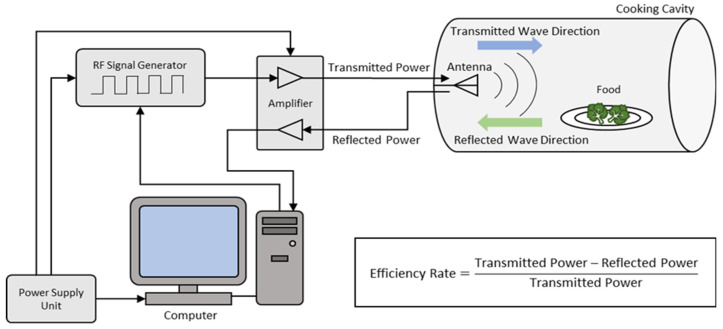
Diagram of the solid-state microwave cooking setup.

A lab-scale solid-state microwave cooking oven is shown in Figure 1.

The experimental setup included a cooking cavity, a vector signal generator (Keysight N5172B, EXG, 9 kHz–6 GHz, Santa Rosa, CA, USA), a solid-state RF power amplifier (IFI SMV350, 500 MHz–1 GHz, Reinach, Switzerland), a high-quality coaxial cable, an antenna, and a computer algorithm that allowed for the control and monitoring of the forward and reflected power.

The optimum frequency within the 902–928 MHz range was scanned for each food separately. The microwave signal at the determined frequency was generated by the signal generator, with the amplitude adjusted to achieve the desired power output. This signal was amplified to 350 W by a power amplifier and transferred to the antenna via a coaxial cable. The antenna then transmitted the signal into the cooking cavity, and the efficiency rate was calculated using the following formula:

(1) $$Efficiency\;rate\;=\;\frac{Power\;transmitted\;into\;the\;cavity\;\left(W\right)\;-\;Reflected\;power\;from\;the\;cavity\;\left(W\right)}{Power\;transmitted\;into\;the\;cavity\;\left(W\right)}$$

### 2.3. Preparation and Cooking of Vegetables

The preparation methods and cooking parameters for the vegetables were similar to those used in previous studies [7,8]. Each vegetable sample was prepared in 100 g portions for the experiments. Broccoli was used in floret form (~5 cm), red peppers were cut into small pieces (2 cm × 2 cm) with stems and seeds removed, and carrots were sliced into pieces with a diameter of 3 cm and a thickness of 1 mm. In order to conduct quality tests for all vegetable groups and cooking methods, the target internal temperature of the foods was set at 75 °C. The cooking conditions for each method are shown in Table 1.

### 2.4. Cooking Quality of Vegetables

#### 2.4.1. Weight Loss

At the end of the cooking process, the weight loss of the food was determined using the following formula:

(2) $$\left(\%\right)Weight\;loss\;=\;\frac{Weight\;of\;the\;raw\;sample\;\left(g\right)\;-\;Weight\;of\;the\;cooked\;sample\;\left(g\right)}{Weight\;of\;the\;raw\;sample\;\left(g\right)}\;\times \;100$$

#### 2.4.2. Moisture Contents and pH and Total Soluble Solid

The moisture content of vegetables before cooking was measured using a halogen moisture analyzer (HB43, Mettler Toledo Inc., Columbus, OH, USA). The pH was determined using a digital pH meter (HI-2211, Hanna Instruments, Woonsocket, RI, USA). Total soluble-solid contents were determined at 20 °C with a refractometer and reported as the Brix value.

#### 2.4.3. Color Parameters

The color parameters (CIE-Lab uniform color space) of the vegetable crust were determined using a spectrophotometer (Minolta Co., Tokyo, Japan). The L*, C, and Hue angle values were measured at three different points on the vegetables.

#### 2.4.4. Texture

Textural measurements were performed using a texture analyzer (TA.XTplus100C, Stable Micro System, Godalming, UK) equipped with a 5 kg load cell and a Warner Bratzler Blade Set with a “V” slot blade operating at a speed of 5 mm/s. The firmness (N/mm) and toughness (N/mm·s) were calculated using the instrument’s software (Texture Exponent, Version 6.1.15, Stable Micro System, Surrey, UK) [9,10]. Firmness was measured to evaluate the resistance of the vegetables to deformation, providing insights into their structural integrity after cooking. Toughness was assessed to determine the effort required to cut or chew the vegetables, reflecting their textural properties and ease of consumption.

#### 2.4.5. Total Phenolic Contents

To measure total phenolic contents, 5 g of each sample was mixed with 50 mL of methanol. Each mixture was then placed in a water bath at 40 °C for 60 min. Thereafter, 50 μL of each sample was taken in individual test tubes, after which 3 mL of distilled water, 250 μL Folin Ciocalteu reagent, and 750 μL of 7% K_2_CO_3_ were added to each tube. The tubes were then vortexed and incubated at 25 °C for 8 min. Subsequently, 950 μL of distilled water was added to the tubes, and the tubes were left in the dark for 2 h. Absorbance values at 750 nm were measured using an UV/Vis spectrophotometer (PerkinElmer, Shelton, CT, USA). Total phenolic contents were expressed as the mg gallic acid equivalents/100 g sample on a dry basis [11].

#### 2.4.6. Vitamin C Measurements

The extracted sample was analyzed using an HPLC-PAD (Shimadzu, Kyoto, Japan) at a wavelength of 244 nm. The analysis was performed using a reverse-phase C18 column at 20 °C with a mobile phase of tetrabutylammonium hydrogen sulfate (94.5:5.5, *v*/*v*) at an injection volume of 20 µL and a flow rate of 0.6 mL/min. The ascorbic acid content was quantified based on the calibration curve prepared by the ascorbic acid standard [12].

#### 2.4.7. Lycopene Measurements

Lycopene content was analyzed using the spectrophotometric method. Samples were dissolved in hexane, and absorbance values at 503 nm (A503) were measured using a UV/Vis spectrophotometer (PerkinElmer, Shelton, CT, USA) [13].

#### 2.4.8. β-Carotene

For β-carotene measurement, 1 g portion of the ground sample was mixed with 5 mL of +4 °C acetone. The mixture was left at +4 °C for 15 min with periodic shaking to enhance extraction, then vortexed and centrifuged at 1370 rpm for 10 min. The upper liquid phase was collected. The remaining solid phase was re-extracted with an additional 5 mL of acetone, vortexed, and centrifuged under the same conditions. The supernatants from both extractions were combined and filtered through Whatman No. 42 filter paper. The absorbance of the extract was measured at 449 nm using a UV/Vis spectrophotometer (PerkinElmer, Shelton, CT, USA) [14].

#### 2.4.9. Chlorophyll

Chlorophyll content was measured according to AOAC method [15]. A 1 g sample was homogenized with 400 mg of CaCO_3_ and 20 mL of 85% acetone, then filtered and diluted to 50 mL with acetone. The filtrate was transferred to a separatory funnel, where it was sequentially extracted with distilled water and ether to remove acetone. The ethereal phase containing chlorophyll was adjusted to 50 mL with ether, dried with anhydrous Na_2_SO_4_, and absorbance was measured at 660 and 642.5 nm using UV/Vis spectrophotometer (PerkinElmer, Shelton, CT, USA).

### 2.5. Statistical Analysis

Data were collected from three independent experiments for each vegetable type (broccoli, red pepper, and carrot) and analyzed to determine the mean ± standard deviation. Normality distribution of the data was checked using the Kolmogorov–Smirnov test. For multiple comparisons, analysis of variance (ANOVA) was performed using Minitab 16 Statistical Software for Windows^®^ (Minitab Inc., State Collage, PA, USA). Tukey’s new multiple-range test was employed to analyze the differences between treatments. Statistical significance was expressed at the 0.95 probability level (*p* ≤ 0.05).

## 3. Results and Discussion

### 3.1. Selection of the Optimum Frequency for Solid-State Microwave Cooking of Vegetables

**Figure 2:**
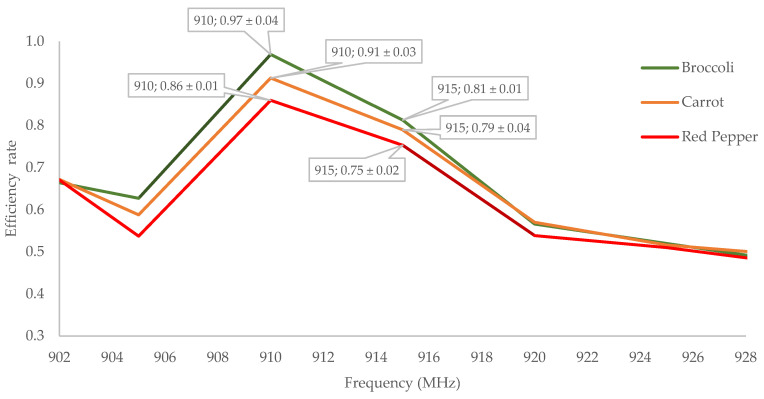
Effect of the operational frequency on the cooking efficiency of different vegetables.

To select the optimum frequency, each of the three food types was individually tested using the solid-state microwave cooking experimental setup designed for this study. Before cooking, the transmitted and reflected power levels were monitored within the 902–928 MHz frequency range (Figure 2).

As illustrated in the graph above, the efficiency of three distinct vegetable types varies with frequency. This phenomenon can be attributed to the dielectric properties of food, which quantify how energy from microwave frequency waves is reflected, stored, or utilized. Factors such as the form, composition, and moisture content of the food significantly influence these properties [16,17]. Although broccoli, red pepper, and carrot differ structurally, they generally belong to the same food category; thus, the trends in efficiency changes relative to frequency variations exhibit notable similarities.

Despite the industry-standard center frequency being 915 MHz, the highest efficiency rate for all vegetable types, as shown in the graph, was achieved at 910 MHz. This finding reflects a significant potential advantage of solid-state microwave technology. This cooking system facilitates the identification of the optimal frequency value for food, allowing for the establishment of appropriate cooking conditions. Therefore, in contrast to existing studies, this research was conducted at a frequency of 910 MHz for solid-state microwave cooking condition.

The cooking times for the processes conducted in accordance with the initially provided experimental design are presented in Table 2.

The longest cooking time was obtained under conventional cooking. However, microwave processing is faster than solid-state microwave cooking, a finding that can be associated with the effect of the higher frequency value and is consistent with the literature [18]. Although longer cooking times are expected to increase nutrient losses, shorter cooking durations, such as those achieved with microwave processing, are anticipated to have a positive effect on the retention of heat-sensitive nutrients, particularly vitamins, when compared to prolonged treatments.

### 3.2. Weight Loss, Moisture Content, pH, and Total Soluble Solids

Weight loss, moisture content, pH, and total soluble solids (TSS) values of samples are given in Table 3. It shows that conventional cooking had the highest weight loss for all three vegetable types and, consequently, the water content observed after conventional cooking was significantly lower compared to other cooking methods (*p* ≤ 0.05).

The water content decreased due to evaporation during cooking. With dielectric methods, a higher water content (versus conventional cooking) may be associated with faster cooking. Dielectric methods use electromagnetic waves and cause water molecules to oscillate, generating heat within the food. This can result in faster cooking times than conventional methods, where heat is transferred primarily through conduction or convection. These results are in agreement with a previous study of ready-to-cook cake and salmon. They indicated that cooking in the range of 800–1000 MHz frequencies provided higher moisture content than conventional cooking [19]. However, another study reported that microwave cooking caused more water loss than other conventional cooking methods because a pressure gradient developed inside the food [20]. Because rapid and effective crust formation does not occur with microwaved food, a higher rate of cooking loss may be observed. On the other hand, solid-state microwave cooking has been observed to result in higher weight loss and lower moisture content compared to traditional microwave cooking. Previous studies have indicated that 915 MHz microwaves possess higher penetration values than those at 2450 MHz, suggesting their effective utilization in drying processes [21]. However, it is important to note that water loss cooking depends on several factors, including the type of food, cooking method, and cooking time [22]. Table 3 shows the pH of raw and cooked vegetables, with microwave-cooked broccoli having the highest pH (6.53) and conventionally cooked red pepper having the lowest pH (5.02). Variations in the pH values of vegetables can be influenced by several factors including the cooking method, cooking duration, and inherent acidity or alkalinity of the vegetables. Previous data showed that conventionally cooked vegetables have the lowest pH among cooked vegetables, indicating a less alkaline environment compared with that of the vegetables cooked by other methods [23]. The higher reduction observed with conventional cooking (compared with microwave and solid-state cooking) is attributable to greater soluble acid degradation and ion concentrations. Similarly, the cooking process caused an increase in the total soluble solid values of red peppers and carrots, particularly under conventional cooking conditions. Furthermore, no significant differences were observed between the two dielectric methods.

### 3.3. Color Assessments

L, C, and Hue angle values of vegetables are listed in Table 4. Colorimetric analysis of raw and cooked samples showed that the different cooking methods significantly influenced the color attributes of the vegetables.

For broccoli, red peppers, and carrots, the L value (an indicator of brightness), decreased with all cooking methods. Microwave treatment resulted in higher brightness values for broccoli and carrots compared to both conventional and solid-state cooking methods. Analysis of the C value revealed a significant increase only in broccoli at the end of the cooking period (*p* ≤ 0.05), likely due to the concentration of specific pigments. The hue angle of broccoli decreased after cooking, indicating a shift from green to yellow, with the most pronounced decrease observed after conventional cooking. This substantial change may be attributed to increased oxidation associated with longer cooking durations. Conversely, no significant change was observed in the hue angle of red peppers. For carrots, the hue angle increased at the end of the cooking period, regardless of the cooking method used. Thorough evaluation of the colorimetric data revealed that the solid-state cooking method generated intermediate results when compared with those obtained using the microwave and conventional cooking methods. This observation is consistent with the results of other physical analyses, suggesting a consistent pattern across various metrics. According to a study on the impact of optimized cooking methods on antioxidant activities in vegetables, microwave cooking preserved the fresh-like color of juice better than other methods [24]. These findings indicate that dielectric treatments may help maintain the original color of vegetables such as broccoli, red peppers, and carrots better than conventional cooking methods.

### 3.4. Texture

The changes in textural properties of foods at the end of cooking are an important quality criterion that affects sensory characteristics. Similar to many other parameters, the changes in texture are specific to each vegetable type. Table 5 presents the toughness and firmness values for different vegetable types before and after cooking.

According to the data presented in the table, significant reductions in both toughness and firmness values were observed for broccoli, red peppers, and carrots post cooking, relative to the initial measurements (*p* ≤ 0.05). Specifically, the solid-state microwave cooking treatment resulted in reductions of 21%, 23%, and 42% for toughness in broccoli, red peppers, and carrots, respectively. In contrast, the percentages recorded after conventional microwave cooking were 14%, 3%, and 41%, whereas conventional cooking yielded reductions of 2%, 14%, and 44%, respectively. The variability in these percentages is intricately linked to the structural characteristics of the food. For instance, the higher toughness value observed under conventional cooking can be attributed to the substantial water loss previously discussed. Conversely, the minimal changes in toughness noted for red peppers during microwave cooking may be ascribed to the shorter cooking durations associated with this method. Moreover, solid-state cooking, especially for broccoli and red peppers, resulted in lower toughness values relative to other cooking methods. As illustrated in Table 5 the firmness values exhibited similar trends. Notably, the most significant overall reductions in both toughness and firmness across all vegetable groups were observed during solid-state cooking.

It is essential to emphasize that achieving similar internal temperatures alone is insufficient to induce textural changes. Conventional cooking, which utilizes an outside-to-center heating approach, can result in significant water loss in foods such as broccoli, leading to less pronounced textural modifications compared to dielectric methods. Conversely, the more substantial changes observed with 910 MHz solid-state microwaves, in contrast to 2.45 GHz microwaves, can be attributed to the effects of the lower frequency. It is well established that lower frequencies are preferred for sterilization in industrial applications due to their higher penetration depth [25]. Cooking vegetables results in the softening of plant tissue, primarily due to the loss of turgor pressure and the breakdown and solubilization of cell wall pectins [9]. Thus, the deeper penetration provided by 910 MHz microwaves may further accelerate these mechanisms, leading to more pronounced textural modifications. In previous studies, contrary to the findings of this research, it has been observed that cooking within the frequency range of 800–1000 MHz offers a significant time advantage over conventional cooking, while not producing a statistically significant difference in texture values [19].

### 3.5. Total Phenolic and Vitamin C Contents

The total phenolic contents in samples are presented in Table 6.

The highest total phenolic contents were found in raw samples, whereas the lowest were measured in vegetables cooked conventionally in all three cases. These results agree with those of researchers who found that combi oven cooking caused a greater decrease in total phenolic compounds in broccoli than microwave cooking [8]. Similarly, it was observed that the phenolic contents of broccoli samples were higher after microwave cooking than after other cooking treatments [26] In contrast, some authors have reported that thermal treatments may cause the decomposition of heat-sensitive phenolic compounds and increase the levels of free flavonols [23,24,25,26,27]. Additionally, another study investigating the efficacy of low-frequency microwave applications for drying compared microwave treatments at 915 MHz and 2450 MHz. In parallel with the results obtained in this research, it was noted that both frequency values yielded similar outcomes regarding total phenolic content [2].

According to the data presented in Table 6, the concentrations of vitamin C in broccoli ranged from 204.85 to 372.99 mg/100 g, while in red peppers the values ranged from 372.43 to 497.74 mg/100 g. In carrots, the concentration was observed to be between 66.52 and 9.17 mg/100 g. These results show that cooking affected the vitamin C content of vegetables, leading to a decline in their nutritional value. The decrease in the vitamin C content upon cooking can be attributed to the oxidation of ascorbic acid, which is accelerated by heat [23,24,25,26,27,28]. Previous data showed that microwaving reduced the processing time, which minimized vitamin C degradation [29]. Similarly, other findings have suggested that microwave cooking is more effective in preserving the vitamin C contents of foods than conventional cooking methods [30,31]. On the other hand, in the comparison of microwave applications, the least reduction in vitamin C levels for broccoli (19.5%) was recorded during microwave cooking, whereas the lowest reduction in vitamin C for carrots (32%) was noted during solid-state microwave cooking. Consequently, it can be inferred that the observed changes are closely associated with the type of food. The potential advantage of 910 MHz for carrots in terms of vitamin C retention may be associated with the deeper penetration of heat, which could help prevent localized overheating. Although further research is needed to confirm this, the higher moisture loss observed in carrots during solid-state microwave cooking compared to microwave cooking suggests that heat distribution may have been more uniform throughout the sample.

### 3.6. Chlorophyll, Lycopene, and β-Carotene Content

The measurement results of chlorophyll, lycopene, and beta-carotene associated with the color pigments of each vegetable group are presented in Table 7. When assessing chlorophyll levels, the lowest change compared to baseline measurements was observed during microwave cooking, with a reduction of 25.6%. This was followed by solid-state microwave cooking, which resulted in a reduction of 35.7%, and conventional cooking, which led to a reduction of 68.2%. The chlorophyll results are highly consistent with the color measurement findings for broccoli. Similarly, it was reported that microwave treatment resulted in less chlorophyll loss in faba bean sauce compared to conventional pasteurization [32].

The level of lycopene, a critical color pigment in red peppers, exhibited a significant decrease at the conclusion of cooking, paralleling trends observed for other nutrients. The lycopene content in raw red peppers was quantified at 90.55 ± 1.74 mg/100 g, while the lowest levels were recorded in conventionally cooked samples (86.48 ± 3.03 mg/100 g). In contrast, microwave-cooked samples demonstrated the highest lycopene content (89.29 ± 0.61 mg/100 g), closely followed by samples prepared using solid-state methods. It was also found that the lycopene content in tomatoes was affected by the cooking method, with different temperatures and durations leading to varying degrees of lycopene retention. Microwave cooking resulted in a higher percentage of retained lycopene than baking or frying [33].

The β-carotene content of carrots increased by approximately 14.7% compared to the initial levels in both microwave applications. In contrast, a decrease of 14.5% was observed compared to the raw carrot under conventional cooking. The findings of this study align with previous research. In investigations examining the variations in β-carotene levels among different vegetable types under various cooking conditions, elevated concentrations of β beta-carotene were observed in foods cooked using microwave methods. The microwave processing of plant materials results in the softening of cell walls, which, in turn, facilitates the disruption of carotenoid–protein complexes, thereby enhancing the extraction of carotenoids. Furthermore, it is hypothesized that this increased extraction may correlate with improved bioavailability [31]. On the other hand, it is known that carotenoids in carrots are located in crystalline chromoplasts surrounded by membranes rich in polar lipids. Thermal processing can alter the physical structure of these carotenoids [34]. In particular, conventional cooking facilitates the solubilization of carotenoids by cellular lipids, as well as leading to the loss of β-carotene due to moisture loss.

## 4. Conclusions

Solid-state microwave cooking is a promising alternative to traditional cooking methods and has demonstrated strong potential for enhancing food quality and nutritional retention. In this study, the effects of solid-state microwave (910 MHz), microwave (2450 MHz), and conventional cooking on the quality of broccoli, carrots, and red peppers were compared, focusing on parameters such as weight loss, moisture content, pH, TSS value, color, texture, total phenolic content, vitamin C, chlorophyll, lycopene, and β-carotene content. The results indicate that solid-state microwave cooking effectively retained moisture content better than conventional cooking methods, which is crucial for preserving the texture and juiciness of vegetables. The reduced moisture loss observed with solid-state and microwave cooking can be attributed to shorter cooking times and more efficient heating mechanisms inherent to dielectric methods. In terms of pH, TSS, and color changes, solid-state and microwave cooking methods resulted in less drastic variations compared to conventional cooking. These findings also demonstrated that solid-state microwave cooking at lower frequencies minimized changes in compounds such as total phenolic content, vitamin C, β-carotene, and lycopene compared to conventional cooking. These results closely resembled those obtained under domestic microwave cooking conditions, further validating the potential for solid-state microwave technology in home appliances. However, the scalability of this technology and its effects on a wider variety of food matrices, particularly in terms of fat and protein content, require further investigation. Therefore, this technology emerges as a promising option for future kitchen appliances, but further research is needed to explore the detailed benefits of this technology and optimize it for various food items.

## Figures and Tables

**Table 1:** The cooking conditions for conventional, microwave, and solid-state microwave cooking.

Cooking Parameters	Cooking Conditions		
	Conventional Cooking	Microwave Cooking	Solid-State Microwave Cooking
Cooking temperature	180 °C	NA	NA
Internal temperature	75 °C	75 °C	75 °C
Frequency	NA	2450 MHz	902–918 MHz ^1^
Power	NA	350 W	350 W
Device model	Arçelik A.Ş., AFM 340 I, İstanbul, Turkey	Arçelik A.Ş., KMF 833, İstanbul, Turkey	NA

^1^ Optimum frequency between 902 and 928 MHz.

**Table 2:** Range of cooking time for each condition.

	Min and Max Cooking Time (min)
**Broccoli**	
Conventional cooking	14–15
Microwave cooking	6–7
Solid-state microwave cooking	9–11
**Peppers**	
Conventional cooking	8–10
Microwave cooking	3–4
Solid-state microwave cooking	6–7
**Carrot**	
Conventional cooking	15–16
Microwave cooking	8–10
Solid-state microwave cooking	11–12

**Table 3:** Effect of cooking method on weight loss, moisture content, pH, and TSS.

	Weight Loss (%)	Moisture Content (%)	pH	TSS (Brix)
**Broccoli**				
Raw	NA	87.32 ± 0.65 ^a^	6.48 ± 0.07 ^a^	1.35 ± 0.84 ^a^
Conventional cooking	31.25 ± 5.24 ^a^	78.94 ± 3.82 ^b^	6.27 ± 0.14 ^b^	1.90 ± 0.55 ^a^
Microwave cooking	11.65 ± 2.64 ^b^	85.14 ± 1.00 ^a^	6.45 ± 0.08 ^a^	1.90 ± 0.22 ^a^
Solid-state microwave cooking	13.18 ± 5.50 ^b^	84.04 ± 2.34 ^a^	6.42 ± 0.07 ^ab^	1.88 ± 0.84 ^a^
**Pepper**				
Raw	NA	90.55 ± 1.74 ^a^	5.08 ± 0.03 ^a^	8.03 ± 0.58 ^b^
Conventional cooking	24.17 ± 3.67 ^a^	86.48 ± 3.03 ^b^	5.01 ± 0.07 ^a^	10.25 ± 0.85 ^a^
Microwave cooking	8.48 ± 0.46 ^b^	89.29 ± 0.61 ^ab^	5.05 ± 0.05 ^a^	8.67 ± 0.47 ^b^
Solid-state microwave cooking	13.17 ± 3.21 ^b^	87.94 ± 2.34 ^ab^	5.07 ± 0.07 ^a^	8.75 ± 0.82 ^b^
**Carrot**				
Raw	NA	87.85 ± 0.40 ^a^	6.59 ± 0.07 ^a^	2.18 ± 0.2 ^b^
Conventional cooking	34.79 ± 1.05 ^a^	80.41 ± 1.62 ^c^	6.09 ± 0.03 ^c^	3.44 ± 0.95 ^a^
Microwave cooking	10.45 ± 1.60 ^c^	86.20 ± 0.71 ^a^	6.36 ± 0.08 ^b^	2.88 ± 0.33 ^ab^
Solid-state microwave cooking	17.04 ± 3.91 ^b^	83.42 ± 2.47 ^b^	6.18 ± 0.12 ^c^	3.08 ± 0.56 ^ab^

NA: Not Applicable. The values shown represent means ± standard deviations. Mean values with different letters (a, b, ab, or c) in the same column are statistically different (*p* ≤ 0.05).

**Table 4:** Effect of the cooking method on the color attributes of vegetables.

	L	C	Hue Angle
**Broccoli**			
Raw	51.96 ± 0.40 ^a^	21.76 ± 1.15 ^b^	111.11 ± 0.71 ^ab^
Conventional cooking	45.59 ± 0.74 ^b^	32.14 ± 2.76 ^a^	102.08 ± 3.33 ^c^
Microwave cooking	47.43 ± 0.79 ^b^	32.66 ± 1.89 ^a^	112.74 ± 1.50 ^a^
Solid-state microwave cooking	46.72 ± 1.78 ^b^	28.77 ± 0.27 ^a^	104.72 ± 3.64 ^bc^
**Peppers**			
Raw	39.74 ± 1.23 ^a^	35.24 ± 1.59 ^a^	36.17 ± 1.86 ^a^
Conventional cooking	38.22 ± 0.13 ^ab^	37.24 ± 2.23 ^a^	40.18 ± 1.50 ^a^
Microwave cooking	37.53 ± 1.02 ^b^	38.68 ± 1.14 ^a^	38.99 ± 1.57 ^a^
Solid-state microwave cooking	38.00 ± 0.31 ^ab^	36.85 ± 2.63 ^a^	36.17 ± 1.86 ^a^
**Carrots**			
Raw	52.05 ± 2.80 ^a^	52.21 ± 4.30 ^a^	52.09 ± 0.31 ^b^
Conventional cooking	46.89 ± 2.30 ^b^	48.52 ± 2.68 ^a^	56.81 ± 1.43 ^a^
Microwave cooking	47.52 ± 0.26 ^ab^	45.51 ± 2.28 ^a^	55.08 ± 1.43 ^a^
Solid-state microwave cooking	46.93 ± 0.69 ^b^	45.82 ± 0.89 ^a^	55.41 ± 1.00 ^a^

The values shown represent means ± standard deviations. Mean values with different letters (a, b, ab, c, or bc) in the same column are statistically different (*p* ≤ 0.05).

**Table 5:** Effect of the cooking method on the texture of vegetables.

	Toughness N/mm.s	Firmness N/mm
**Broccoli**		
Raw	15.10 ± 4.12 ^a^	1.92 ± 0.61 ^a^
Conventional cooking	14.77 ± 2.05 ^ab^	1.89 ± 0.51 ^a^
Microwave cooking	12.94 ± 3.55 ^ab^	1.87 ± 0.99 ^a^
Solid-state microwave cooking	12.01 ± 3.22 ^b^	1.82 ± 051 ^a^
**Peppers**		
Raw	10.79 ± 1.98 ^a^	0.72 ± 0.16 ^a^
Conventional cooking	9.29 ± 3.44 ^ab^	0.62 ± 0.14 ^ab^
Microwave cooking	10.44 ± 3.03 ^ab^	0.72 ± 0.16 ^a^
Solid-state microwave cooking	8.27 ± 2.03 ^b^	0.54 ± 0.17 ^b^
**Carrots**		
Raw	11.07 ± 1.16 ^a^	1.72 ± 0.38 ^a^
Conventional cooking	6.23 ± 1.68 ^b^	0.84 ± 0.75 ^b^
Microwave cooking	6.52 ± 1.56 ^b^	0.82 ± 0.52 ^b^
Solid-state microwave cooking	6.43 ± 1.59 ^b^	0.74 ± 0.39 ^b^

The values shown represent means ± standard deviations. Mean values with different letters (a, b or ab) in the same column are statistically different (*p* ≤ 0.05).

**Table 6:** Effect of the cooking method on total phenolic content and vitamin C.

	Total Phenolic Content (mg GAE/100 g Dry Basis)	Vitamin C (mg/100 g Dry Basis)
**Broccoli**		
Raw	146.71 ± 11.59 ^a^	372.99 ± 31.86 ^a^
Conventional cooking	86.25 ± 16.15 ^c^	202.08 ± 54.60 ^c^
Microwave cooking	116.99 ± 14.34 ^b^	300.11 ± 50.00 ^b^
Solid-state microwave cooking	113.34 ± 15.45 ^b^	213.29 ± 27.25 ^c^
**Peppers**		
Raw	168.52 ± 14.12 ^a^	522.11 ± 33.32 ^a^
Conventional cooking	116.51 ± 22.08 ^b^	278.68 ± 67.92 ^c^
Microwave cooking	120.92 ± 25.41 ^b^	409.59 ± 59.96 ^b^
Solid-state microwave cooking	122.91 ± 18.18 ^b^	381.63 ± 38.40 ^b^
**Carrots**		
Raw	107.01 ± 13.62 ^a^	66.52 ± 9.12 ^a^
Conventional cooking	61.58 ± 9.08 ^c^	9.17 ± 3.57 ^d^
Microwave cooking	88.04 ± 11.62 ^b^	28.43 ± 5.88 ^c^
Solid-state microwave cooking	76.14 ± 12.00 ^bc^	45.19 ± 6.86 ^b^

The values shown represent means ± standard deviations. Mean values with different letters (a, b, c, bc, or d) in the same column are statistically different (*p* ≤ 0.05).

**Table 7:** Effect of the cooking method on chlorophyll, lycopene, and β-carotene content.

	Chlorophyll Content for Broccoli (mg/100 Dry Basis)	Lycopene Content for Red Pepper (mg/100 g Dry Basis)	β-Carotene Content for Carrot (mg/100 g Dry Basis)
Raw	910.0 ± 58.7 ^a^	408.35 ± 33.56 ^a^	757.65 ± 67.99 ^b^
Conventional cooking	289.3 ± 58.4 ^d^	224.73 ± 29.19 ^c^	647.52 ± 46.72 ^c^
Microwave cooking	674.0 ± 54.0 ^b^	289.55 ± 21.31 ^b^	866.43 ± 59.76 ^a^
Solid-state microwave cooking	594.4 ± 51.5 ^c^	242.94 ± 28.05 ^bc^	868.69 ± 31.61 ^a^

The values shown are represent means ± standard deviations. Mean values with different letters (a, b, c, bc, or d) in the same column are statistically different (*p* ≤ 0.05).

## Data Availability

The original contributions presented in this study are included in the article. Further inquiries can be directed to the corresponding author.
